# Optical Characterization of Doped Thermoplastic and Thermosetting Polymer-Optical-Fibers

**DOI:** 10.3390/polym9030090

**Published:** 2017-03-04

**Authors:** Igor Ayesta, María Asunción Illarramendi, Jon Arrue, Itxaso Parola, Felipe Jiménez, Joseba Zubia, Akihiro Tagaya, Yasuhiro Koike

**Affiliations:** 1 Department of Applied Mathematics, University of the Basque Country (UPV/EHU), Engineering School of Bilbao, Plaza Ingeniero Torres Quevedo, 1, E-48013 Bilbao, Spain; felipe.jimenez@ehu.eus; 2 Department of Applied Physics I, University of the Basque Country (UPV/EHU), Engineering School of Bilbao, Plaza Ingeniero Torres Quevedo, 1, E-48013 Bilbao, Spain; ma.illarramendi@ehu.eus (M.A.I.); itxaso.parola@ehu.eus (I.P.); 3 Department of Communications Engineering, University of the Basque Country (UPV/EHU), Engineering School of Bilbao, Plaza Ingeniero Torres Quevedo, 1, E-48013 Bilbao, Spain; jon.arrue@ehu.eus (J.A.); joseba.zubia@ehu.eus (J.Z.); 4 Faculty of Science and Technology, Keio University, 3-14-1 Hiyoshi, Kohoku-ku, Yokohama 223-0061, Japan; a-tagaya@kpri.keio.ac.jp (A.T.); koike@appi.keio.ac.jp (Y.K.)

**Keywords:** light-emitting polymers, polymer optical fibers, thermoplastic fibers, thermosetting fibers, amplified spontaneous emission, optical gain, rhodamine 6G

## Abstract

The emission properties of a graded-index thermoplastic polymer optical fiber and a step-index thermosetting one, both doped with rhodamine 6G, have been studied. The work includes a detailed analysis of the amplified spontaneous emission together with a study of the optical gains and losses of the fibers. The photostability of the emission of both types of fibers has also been investigated. Comparisons between the results of both doped polymer optical fibers are presented and discussed.

## 1. Introduction

The last few years have seen a remarkable interest in the field of research dealing with the embedding of functional materials in adequate hosts, such as polymer optical fibers (POFs), in order to fabricate solid-state organic amplifiers and lasers [1,2]. In this respect, there exist several well-known advantages of POFs over glass fibers. The manufacturing technology of POFs is cheaper and simpler than that of glass fibers, and their mechanical strength is also higher. Their lower manufacturing temperatures make it possible to dope the fiber core with an ample variety of active materials like organic dyes, conjugated polymers, rare-earth ions, noble-metal nanoparticles, or quantum dots [3,4,5]. High optical gains can be achieved in remarkably short fiber lengths by doping POFs with organic dyes having high absorption and emission cross sections. Furthermore, as compared with the bulk material, the waveguide geometry of optical fibers make them advantageous in that they provide optical confinement in the fiber core, long interaction distances between light and active material, and high volume-to-surface-area ratios. This makes heat dissipation more efficient and, hence, minimizes thermal degradation. As compared with the bulk material, the fiber structure also provides a symmetric output beam profile and straightforward connectivity with the presently existing fiber-optic communications systems. All these advantages make doped POFs a very interesting option to realize efficient amplifiers, lasers, illuminators, sensors, and all-optical switches in the visible region [6,7,8,9].

Since the discoveries of the fiber laser based on a dye-doped POF in 1987 [6] and of the first amplifier based on POFs in 1993 [7], POFs doped with organic dyes have been extensively studied [8,10,11]. Light amplification along the fibers can be achieved just by pumping the doped fibers either longitudinally or transversely. In this type of configuration, the gain is due to amplified spontaneous emission (ASE). ASE presents some laser-emission properties, such as a narrowed emission spectrum, the existence of a threshold-like energy, and directionality, but its output bandwidth is broader than that obtained from a typical laser. These properties make doped POFs suitable for their use as broadband fiber sources. More specifically, these fiber sources, either glass or polymer ones, are nowadays candidates for a large variety of applications, e.g., for spectroscopy, for low-coherence interferometry, for fiber gyroscopes, and for applications that need a broad spectral gain, such as the amplification of all the channels in a wavelength-division-multiplexing system [12,13,14]. One of the most recent research lines regarding broadband POF sources has been the incorporation of multiple laser dyes in the POF so that a single fiber can cover a broad wavelength range [15]. This broad spectrum has been employed in combination with sensing techniques, such as the use of Bragg gratings inscribed in the same fiber, in order to manufacture all-polymer-fiber sensors. This technique makes it possible to interrogate multiple Bragg gratings by using the light generated in the same fiber [16].

As far as we know, the host matrix used in the reported works on dye-doped POFs has always been a thermoplastic polymer, such as polymethylmethacrylate (PMMA). Thermoplastic polymers present a plastic-elastic behavior and they can be melted and reshaped when they are heated. In contrast, thermosetting polymers do not melt upon heating, and therefore they cannot be reshaped to any extent. Thermosetting polymers are made of polymer chains that cross-link with each other irreversibly, thus forming a three-dimensional interconnected polymer structure. The thermal stability of cross-linked polymers is considerably higher than that of non-crossed-linked ones, which could be an important feature if these materials are to be employed for POFs in high-temperature environments [17].

Taking the aforementioned applications of doped POFs into account, we present a thorough experimental study of the fluorescence and ASE properties of a graded-index (GI) thermoplastic POF and of a step-index thermosetting POF doped with rhodamine 6G (R6G) with different dopant concentrations and distributions. R6G is one the most efficient fluorescent dyes, it has been extensively and successfully employed in dye lasers, and it is still being studied nowadays. We analyze the influence of dye distribution, dye concentration, and host matrix on the light emission, which may be useful in improving the performance of broadband light sources based on these types of doped POFs. POFs of identical dye distributions and concentrations would facilitate the analysis of the effect of the host matrix alone. A theoretical study of the influence of the dye distribution on the emission properties of thermoplastic fibers was carried out by the authors in [18]. Regarding thermosetting fibers, there are no previous studies of dye-doped thermosetting POFs in the literature, in spite of their interesting thermal properties. Specifically, we show the absorption and emission bands, the amplified spontaneous emission and the optical gains for both types of fibers using the variable-stripe-length (VSL) method [19]. We also calculate the optical losses for both fibers by using the side-illumination technique [20,21]. Besides, since the potential applications of the studied doped POFs depend on the photodegradation of the dye, we will present the evolution of the emitted fluorescence characteristics when the POFs are excited for prolonged exposure times.

This paper is organized as follows. The employed experimental techniques and the characteristics of the analyzed fibers are described in Section 2. The light amplification properties, the attenuation and the photostability of the two types of POFs are analyzed and discussed in Section 3. The conclusions are summarized in Section 4.

## 2. Experiment

**Figure 1:**
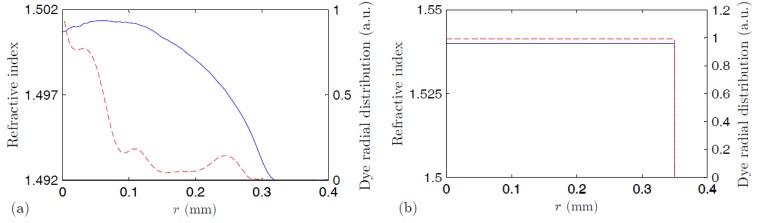
Refractive-index profiles (blue solid lines) and dye radial distributions (red dashed lines) in the fiber core obtained for the thermoplastic POF (**a**) and for the thermosetting POF (**b**).

The samples analyzed are a thermoplastic POF and a thermosetting one, both of them doped with R6G in their cores but not in their claddings. Figure 1 shows the refractive-index profile and non-uniform dopant concentration of the thermoplastic fiber. In order to obtain a graded-index distribution in this fiber, the interfacial gel polymerization technique was employed [22,23]. In this process, a mixed solution of molecules of MMA monomer, polymerization initiator, chain transfer reagent, grading-index reagent (such as triphenyl phosphate), and also rhodamine 6G was prepared. Then, the solution was poured into a PMMA tube and subjected to a polymerization process. During this process, the polymerization took place inward from the wall of the tube. As the molecular volume of the MMA monomer is smaller than that of the grading-index reagent, MMA molecules can diffuse into the polymer phase more easily than the grading-index reagent, so this is gradually concentrated in the center region as the thickness of the polymer phase increases. Since the refractive index of the reagent molecules is higher than that of MMA, a rod with a graded-index distribution is obtained. The refractive-index distribution of the rod was measured by means of the longitudinal interferometric technique [23]. The resulting distribution of the dopant, shown in Figure 1a, was obtained by measuring the absorption from points along the diameter of a disc-shaped sample that was cut out and polished from the preformed rod, using the light from a He-Ne laser at 632.8 nm. From the value of the absorption cross section at this wavelength, the concentration of dopant could be estimated. The radial dye distribution in the POF is almost the same as that of the preform rod [24]. On the other hand, the thermosetting POF is a step-index fiber made of acrylic monomers whose dopant concentration is uniform (see Figure 1). POFs that are graded-index, as is the case of our thermoplastic sample, usually have higher dopant concentrations near the fiber symmetry axis, where the majority of the propagating light is concentrated. Therefore, the interaction between light and dopant molecules in that area is stronger than in the case of step-index fibers, such as our thermosetting sample. A parameter that measures the amount of interaction between dye molecules and light is the so-called overlapping factor γ, which is 1 in the case of step-index fibers and larger than 1 in the case of graded-index fibers (γ ≈ 1.4 in our thermoplastic sample) [18]. The thermoplastic POF was produced by the Keio Photonics Research Institute of Keio University [22,23,24,25], while the thermosetting POF was manufactured by the company Intellisiv Ltd. (Gadera, Israel) [26,27]. None of the fibers are commercially available. Their main manufacturing characteristics are summarized in Table 1. The average concentration of the thermoplastic fiber (16 ppm) is close to the maximum that can be dissolved satisfactorily in the PMMA host, in the sense that higher concentrations would produce detrimental dye aggregates in the fiber [28].

**Figure 2:**
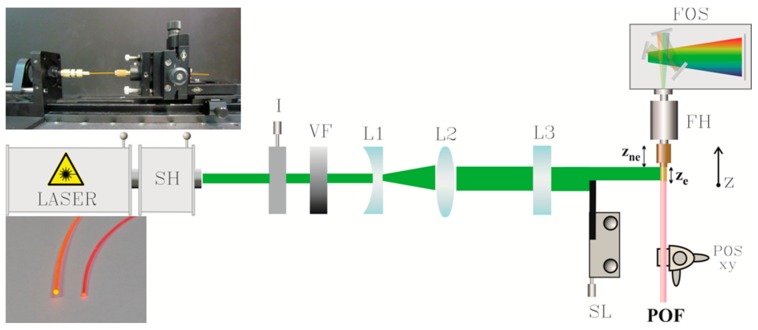
Experimental set-up for the measurement of the ASE and the fiber gain using the VSL method. Legend: SH: second harmonic generator; I: iris; VF: variable filters; L1: divergent lens; L2: convergent lens; L3: cylindrical lens of *f*´ = +15 cm; SL: sliding blade; POSxy: micro positioner; FH: filter holder; FOS: fiber-optic spectrometer. Top inset: photograph of a fiber in the experimental set-up. Bottom inset: photograph of the thermoplastic fiber (**left**) and of the thermosetting one (**right**).

Figure 2 sketches the experimental set-up employed to measure the emission from the fiber upon transverse excitation. For the study of the ASE, the samples were pumped optically with the output signal of a frequency doubler coupled to a Nd:YAG nanosecond laser (EKSPLA NL301HT) (10 Hz, λ = 532 nm). The measurements of the attenuation and of the photostability were carried out by pumping the fibers with a tunable visible femtosecond laser at 80 MHz (Spectra-Physics Mai Tai HP, Newport, Santa Clara, CA, USA) combined with a frequency doubler (Inspire Blue, Radiantis, Barcelona, Spain). A cylindrical lens (L3) was used to convert the circular beam spot into a narrow stripe, which was focused onto the fiber side. The illuminated area was 1 mm in height and *z_e_* mm in width. For the measuring of the optical gains of the fibers, the VSL method was employed in the range of *z_e_* between 0 and a certain maximum length, *z_e,max_* [29]. In all our measurements, the emitted light propagates through a non-excited length *z_ne_* before reaching the detector. A fiber-optic spectrometer (Ocean Optics USB4000, with an optical resolution of 1.5 nm of full width at half maximum) was used for the spectral measurement of the emission from one of the fiber ends. The absorption spectra of the doped fibers were obtained with a Cary 50UV–VIS spectrophotometer (Agilent Technologies, Santa Clara, CA, USA) equipped with a fiber-optic coupler accessory.

## 3. Results and Discussion

### 3.1. Absorption, Fluorescence, and ASE Spectra

**Figure 3:**
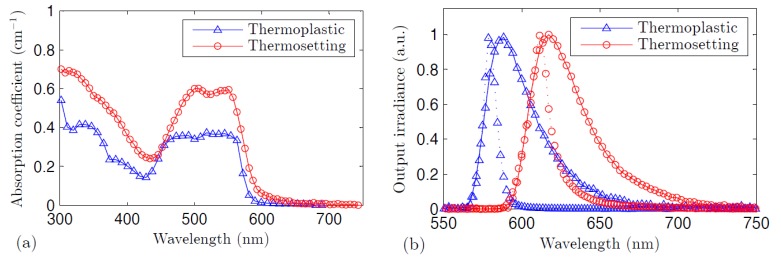
(**a**) Absorption coefficients corresponding to the thermoplastic POF and to the thermosetting POF; (**b**) Fluorescence spectra (solid lines) and ASE spectra (dotted lines) corresponding to both types of POFs. The fluorescence excitation irradiances are 0.1 mJ·cm^−2^ (0.01 mJ of pump energy with *z_e_* = 0.92 cm) for the thermoplastic POF and 2.88 mJ·cm^−2^ (0.265 mJ with *z_e_* = 0.92 cm) for the thermosetting POF. The ASE excitation irradiances are 41.3 mJ·cm^−2^ (3.8 mJ with *z_e_* = 0.92 cm) and 72 mJ·cm^−2^ (6.6 mJ with *z_e_* = 0.92 cm), respectively.

The absorption spectra of our two types of R6G-doped POFs are shown in Figure 3a. In both cases, there is a strong wide absorption band that extends in the green region of the spectrum, corresponding to the transition from the ground singlet state S_0_ to the excited singlet state S_1_. As also happens in some other R6G-doped PMMA systems, the absorption bands present a flattened shape due to the contributions of dye monomers and aggregates [30]. A much weaker absorption band is detected around 350 nm, which is only noticeable in the thermoplastic POF, since, in the thermosetting fiber, it is hidden by the ultraviolet tail of the absorption curve of the host material. This weaker band corresponds to the transition from S_0_ to the excited singlet state S_2_. Notice that the absorption spectrum of the thermosetting fiber is located at slightly longer wavelengths than that of the thermoplastic fiber. The values of the attenuation coefficients at wavelengths outside the absorption band of the dye most probably stem from the contribution of light scattering in the fiber core. Since the lengths of our measured fibers are very short (about 1 cm), the power mode distributions of the fibers have not yet reached the equilibrium condition, so the attenuation coefficients at those wavelengths may be higher than those obtained by using long fibers, as demanded by the standard cut-back method. Figure 3b shows the fluorescence and ASE spectra when the fibers are excited transversely at 532 nm, which correspond to the transition from the excited state S_1_ to the ground singlet state S_0_. The ASE spectra, obtained by pumping the fibers above the threshold value, are the narrowed bands in the figure. It can be observed that both the fluorescence and the ASE curves of the thermosetting POF, with a higher dopant concentration, are located at longer wavelengths (about 30 nm) than the corresponding curves of the thermoplastic POF, with a lower dopant concentration (see Table 2). Additionally, it can be observed that the fluorescence spectrum of the thermosetting POF is broader than that of the thermoplastic POF. This spectral location at longer wavelengths and the broader spectrum in the case of the thermosetting POF could be explained by its higher dye concentration. It is known that dopant molecules tend to link to one another when the dye concentration is higher than a certain maximum level, thus creating dopant aggregates and, consequently, making the emission bands shift toward longer wavelengths [31]. In this respect, the crosslinked chains of the thermosetting polymer favor the appearance of dye aggregates when the dopant concentration is high enough [32].

**Figure 4:**
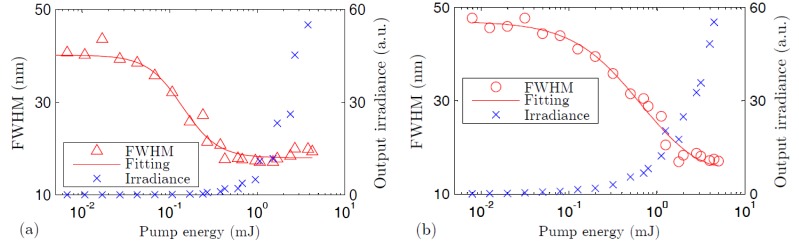
Spectral full width at half maximum and irradiance of the light emitted from the thermoplastic POF (**a**) and from the thermosetting POF (**b**). In both fibers, *z_e_* and *z_ne_* were maintained constant (*z_e_* = 0.92 cm and *z_ne_* = 3.3 cm).

**Figure 5:**
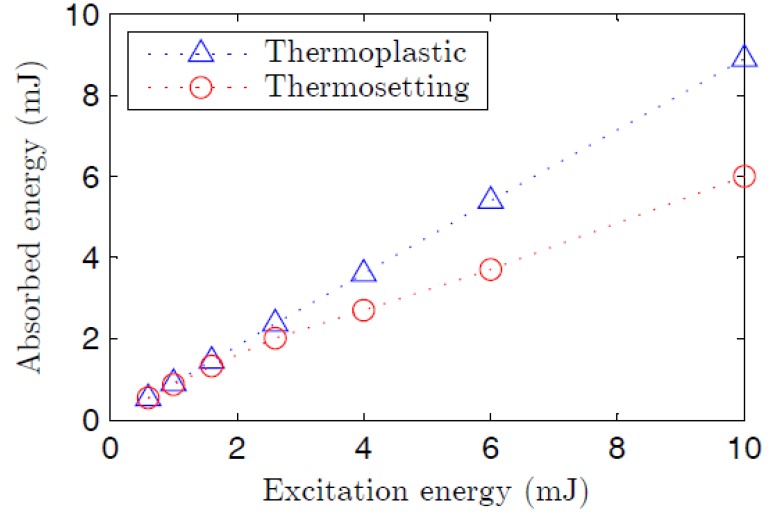
Experimentally-obtained values of the energy absorbed in each fiber as a function of the incident pump energy when *z_e_* = 0.92 cm. The dashed lines serve to guide the eye.

Figure 4 illustrates the spectral narrowing and the abrupt increase in the output irradiance that occurs when the pump energy becomes higher than the ASE threshold. Due to gain-saturation effects, the spectral widths never become narrower than a certain value, and the curves of the full width at half maximum (FWHM) tend to become rather horizontal when the pump energy is high enough. The ASE threshold has been estimated as the pump energy at which the spectral FWHM of the emission spectrum decays to half of its maximum value [33]. As can be observed in the figure, the threshold is higher in the case of the thermosetting fiber (see Table 2 for the exact values). This fact could be due, in part, to the lower pump energy that is absorbed by the thermosetting fiber, as shown in Figure 5, where it is also noticeable that there is a greater tendency for absorption saturation at high pump energy to occur in this fiber, possibly due to phenomena such as excited-state absorption, energy transfer to triplet states, and so on. On the other hand, the aggregation of dye molecules at high concentration—such as that of the thermosetting POF—reduces the effective surface area of the dopant and hence its absorption cross section. This reduction has been attributed to the mutual interaction of neighboring molecules [34]. Besides, the fact that the overlapping factor *γ* is smaller and the core diameter is greater in our thermosetting POF could also contribute to its higher threshold energy [18].

### 3.2. Optical Gain

**Figure 6:**
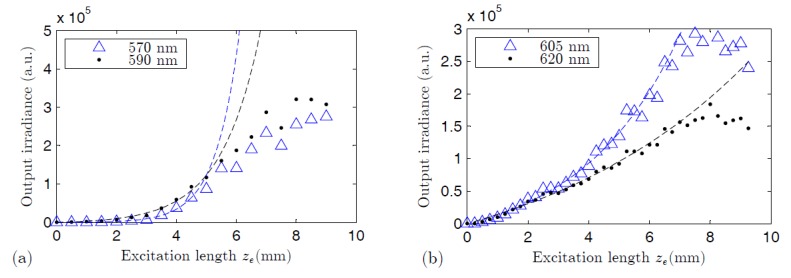
Output irradiance from the thermoplastic POF (**a**) and from the thermosetting POF (**b**) measured at two different emission wavelengths in each case: 570 and 590 nm in the case of the thermoplastic fiber, and 605 and 620 nm in the case of the thermosetting fiber one. The pump irradiances are 6 and 30 mJ·cm^−2^, respectively. *z_e,max_* = 0.92 cm in both cases. The dashed lines are the fittings using Equation (1).

**Figure 7:**
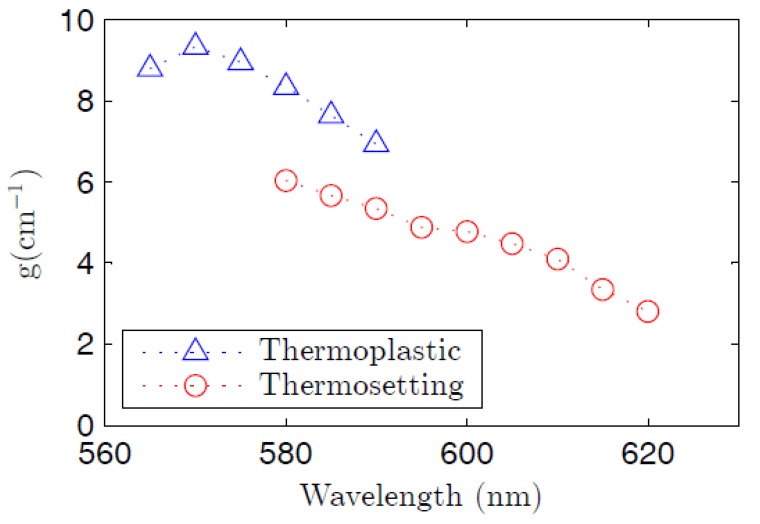
Spectral gains of the thermoplastic fiber and the thermosetting one. The pump irradiances are 6 and 30 mJ·cm^−2^, respectively.

The gain coefficient of each fiber is calculated by measuring the output irradiance at the desired wavelength *λ* as a function of the excitation length *z_e_* (VSL method). In Figure 6a,b the output ASE intensities from the two types of POFs have been plotted as functions of *z_e_* for two different wavelengths in each case. As can be seen, the irradiance curves grow in an approximately exponential way with the excitation length. In each case, the gain coefficient at the desired emission wavelength can be estimated by fitting the irradiance, for a range of values *z_e_*, to the following equation:

(1) $$I(\lambda ,z_{e})=\frac{C\left(\lambda \right)}{g\left(\lambda \right)}\left[\exp\left(g\left(\lambda \right)z_{e}\right)-1\right],$$

where *C*(*λ*) is a constant related to the spontaneous emission and *g*(*λ*) is the gain coefficient at the emission wavelength considered [30]. The disagreement between the fitted curves and the experimental points for the largest values of *z_e_* may be due to saturation effects. The gains have been calculated in a range of wavelengths, and the corresponding curves obtained for both fibers are represented in Figure 7. In spite of the greater pump irradiances employed with the thermosetting fiber, the gains are quite similar in both fibers, or even slightly greater in the thermoplastic one. This gain reduction in our thermosetting POF could be explained, again, by taking the greater presence of dye aggregates in its strongly doped fiber core into account. These aggregates cause a negative effect by reducing the fluorescence efficiency and by decreasing the stimulated emission cross section of the dye [28,35]. On the other hand, the lower value of the overlapping factor *γ* in the step-index thermosetting POF also tends to reduce the gain coefficients [18]. The gains shown in Figure 7 are on the same order of magnitude as those reported for electrospun R6G-doped PMMA nanofibers [36].

### 3.3. Optical Loss

**Figure 8:**
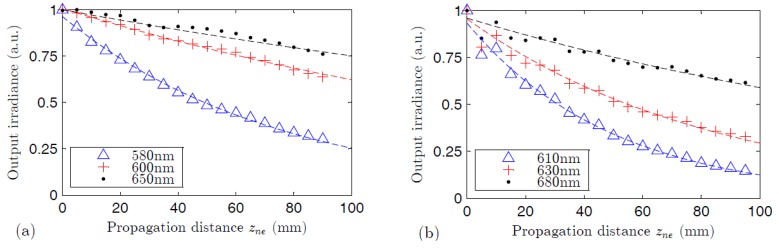
Output irradiance as a function of the propagation distance for the thermoplastic POF (**a**) and for the thermosetting POF (**b**) at three different emission wavelengths: 580, 600, and 650 nm for the thermoplastic fiber, and 610 , 630, and 680 nm for the thermosetting fiber. The dashed lines are the fittings to Equation (2). The excitation wavelength in both cases is 520 nm with 22 nJ·cm^−2^ of irradiance.

The attenuation in both fibers has been calculated by measuring the decrease of the irradiance of the fluorescence spectra as the light propagation distance (*z_ne_*) is increased with a constant excitation length (*z_e_* = 1.2 mm). In Figure 8, we can see how this irradiance decreases for three different emission wavelengths. Assuming that the illuminated fiber portion behaves as a plane-wave source, the light output measured at the fiber end at the emission wavelength decays exponentially [20]:

(2) $$I(\lambda ,z_{ne})=I_{0}\left(\lambda \right)\exp\left(-\alpha \left(\lambda \right)\cdot z_{ne}\right),$$

**Figure 9:**
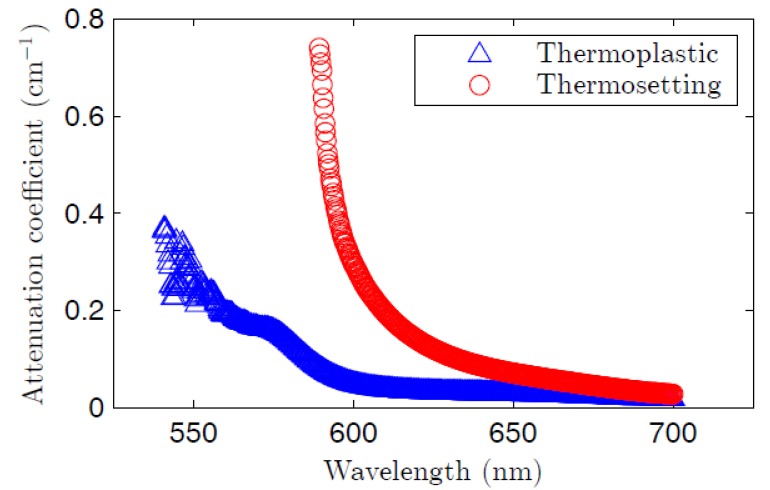
Linear-attenuation coefficient for both the thermoplastic fiber and the thermosetting one. At 700 nm, this coefficient is 0.018 cm^−1^ in the thermoplastic fiber and 0.027 cm^−1^ in the thermosetting one. The excitation irradiance at 520 nm is 22 nJ·cm^−2^.

In Equation (2), *I*_0_(*λ*) is the light irradiance measured at *z_ne_* = 0 at a certain wavelength *λ*, and α(*λ*) is the linear attenuation coefficient at that wavelength. Figure 9 shows the linear-attenuation coefficients obtained for the two fibers by fitting experimental curves *I*(*λ*,*z_ne_*) for several wavelengths to Equation (2) [20,37]. In agreement with Figure 3a, the highest values of the attenuation curve in the range of wavelengths considered correspond to the thermosetting POF. In the region where the dye absorption tends to be negligible, i.e., above 650 nm, the attenuation of the thermosetting fiber is slightly higher. This result could be due to the light-scattering effect of the dye aggregates, and also to the fact that host materials with higher melting temperatures, as is the case of the thermosetting host, tend to present higher propagation losses [17,38]. Also in the case of non-doped fibers, the attenuation coefficient of undoped thermosetting POFs is typically higher than that of undoped PMMA POFs (on the order of 1500 and 400 dB/km, respectively, at 700 nm) [2,39]. In both types of fibers, the attenuation coefficients are always much smaller than the gain coefficients (see Figure 7).

**Figure 10:**
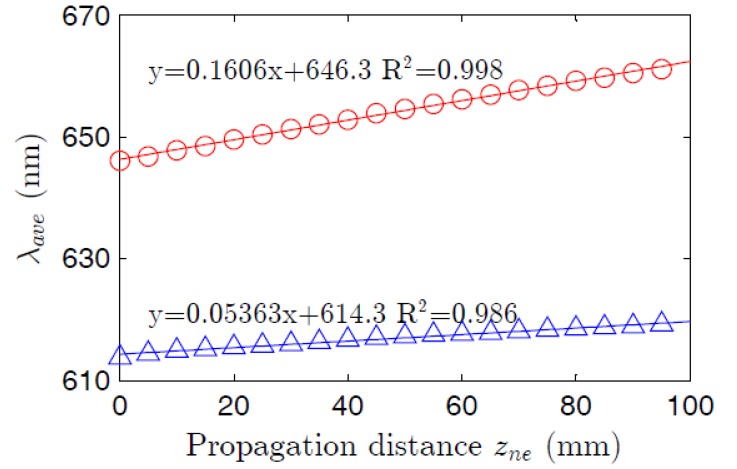
Evolutions of the average wavelengths of the emission spectra of the thermosetting fiber (o) and the thermoplastic fiber (Δ) as functions of the propagation distance. The pump irradiance was 22 nJ·cm^−2^ at 520 nm. The equations correspond to the linear fittings (solid lines).

The average emission wavelengths of the fluorescence curves in Figure 10 are shifted linearly toward longer wavelengths as *z_ne_* is increased, which is due to reabsorption and reemission effects [37]. Figure 10 shows this shift for both types of fibers. The curves in Figure 10 present a linear behavior, but with different slopes: 0.054 nm/mm for the thermoplastic POF and 0.16 nm/mm for the thermosetting one. This linear spectral shift with length enables the manufacture of tunable light sources, and it could also be useful for the design of displacement sensors using doped fibers. For this purpose, the greater slope of the thermosetting POF would be advantageous.

### 3.4. Photostability

**Figure 11:**
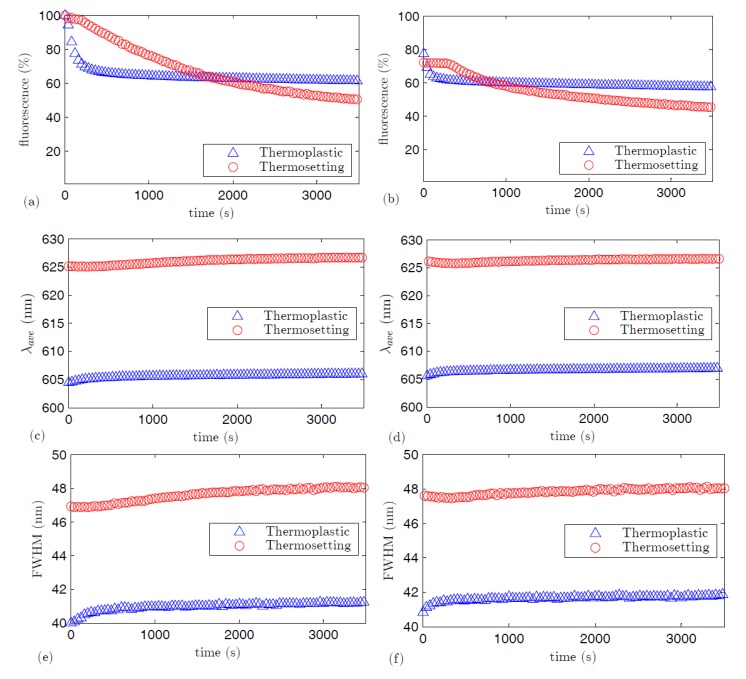
Fluorescence intensities, (**a**) and (**b**); average wavelengths, (**c**) and (**d**); and FWHMs (**e**) and (**f**), as functions of the excitation time. The excitation wavelength was 520 nm, the excitation irradiance was 22 nJ·cm^−2^, and the light propagation distance was 4.4 cm. The fibers were excited for 60 min in (**a**), (**c**), and (**e**). The fibers were re-excited for another 60 min after a 24 h rest in (**b**), (**d**), and (**f**).

The photostability of the fluorescence spectra has been studied for both fibers with *z_e_* = 0.12 cm and *z_ne_* = 4.4 cm by pumping them at 520 nm with the femtosecond laser. In a first step, both fibers were exposed to the same excitation conditions for 60 min The irradiance, average wavelength, and FWHM of the fluorescence spectra obtained during this period of time are shown in Figure 11a,c,e. In a second step, the same magnitudes were measured under the same conditions for another 60 min after a rest of 24 h in darkness, and the corresponding results have been plotted in Figure 11b,d,f. In the first step, the fibers had never been exposed to laser light, so there was no previous degradation that could affect the results. For the sake of comparison, we have normalized the fluorescence intensities to 100% at the start of the first set of measurements.

As can be noted in Figure 11a, the rhodamine-6G-doped thermoplastic fiber is more stable than the thermosetting one. Specifically, after 60 min of exposure, which, in this case, is equivalent to 2.88 × 10^11^ laser shots, the fluorescence capacities of both fibers were reduced by 39% and by 50%, respectively. According to the definition of lifetime given in [40], which is the time elapsed before the fluorescence irradiance is reduced by half, the thermoplastic fiber does not reach its lifetime value in the first set of measurements, whereas the thermosetting fiber reaches it just at the end of the same excitation period. The decays of the emitted fluorescence shown in Figure 11a can be well fitted to a double-exponential expression, where *a*, *b*, *τ*_1_, and *τ*_2_ are constants:

(3) $$f(t)=a\exp(-t/\tau_{1})+b\exp(-t/\tau_{2}),$$

The values of these constants were obtained from the fittings, and the corresponding coefficients of determination *R*^2^ are shown in Table 3. The values of *τ*_1_ and *τ*_2_ shown in Table 3 corroborate that the thermoplastic fiber undergoes the fastest degradation at the beginning of the excitation period, but the steady state is achieved relatively fast. Specifically, the thermoplastic fiber achieves an approximately steady state after 5 min of exposure, as can be seen in Figure 11a. In the case of the thermosetting fiber, the fluorescence irradiance is not much affected during the first 3 min, maintaining its emission capacity almost at 100%. However, after this short excitation period, the emission irradiance diminishes steadily during the rest of the excitation time without reaching any steady state.

The observed photodegradation that these fibers undergo could be due to different mechanisms, whose influence is different depending on the fiber. The most common source of degradation in dye-doped polymers is the thermally-induced bleach of organic dyes. In this respect, in our thermoplastic fiber, the dopant molecules are mainly surrounded by the host matrix, due to the lower dye concentration. Consequently, the molecules are partially protected from the thermally-induced degradation effects, and the fiber conserves its emission capacity for a longer time [40]. There are also other factors in our thermoplastic fiber that contribute to a better photostability, such as the lower presence of dye aggregates in the host matrix. The presence of aggregates would tend to increase the number of non-radiative relaxations and, therefore, would tend to contribute to the optical bleaching of the dye, as probably happens in our thermosetting POF [10,28]. Another disadvantage of our thermosetting POF, which has a step-index profile, is that step-index fibers tend to be less photostable than, for example, hollow fibers or graded-index fibers [41]. This fact probably contributes to the lower degradation of our graded-index thermoplastic fiber.

The emission spectra change slightly with excitation time, as shown in Figure 11c,e. We can see that, in both types of fibers, there is only a nearly negligible red shift in the average wavelength and a slight spectral broadening in the range of exposure times considered. Similarly, spectral changes with exposure time were also observed in active POFs doped with other dopants [42].

As already commented, we have also analyzed the effects of excitation after a rest period of 24 h in darkness. As Figure 11b shows, there is a partial recovery in the fluorescence capacity during the rest time both in the thermoplastic POF and in the thermosetting one: the emission intensities are, respectively, 16% and 22.3% higher right after the rest. This partial recovery has also been reported for other types of doped fibers and thin films, which suggests that there exist some reversible photodegradation processes [43]. Despite the partial recovery in the emission intensities during the rest time in both types of fibers, the changes in the emission average wavelength and in the spectral width are insignificant with respect to the values obtained just before the rest (see Figure 11d,f).

## 4. Conclusions

We have compared the emission properties of a graded-index thermoplastic POF and of a step-index thermosetting POF, both doped with rhodamine 6G, but with different distributions and concentrations: low concentration and non-uniform distribution in the thermoplastic POF, and high concentration and uniform distribution in the thermosetting POF. The properties of the amplified spontaneous emission, the optical losses, and the photostability of the emission have been analyzed for each type of fiber by using transverse excitation. We have found that the ASE characteristics, such as the threshold value and the spectral optical gains, are slightly better in the thermoplastic fiber. Besides, the attenuation of the generated emission is lower in the thermoplastic fiber. An analysis of the photostability carried out for both fibers shows that the thermoplastic fiber is more stable than the thermosetting one. The most influential factors in the emission properties presented in this work are the concentration and the distribution of the dye, the influence of the host matrix being much smaller. The much higher concentration in the thermosetting POF causes some negative effects on its emission properties due to the presence of aggregates. On the other hand, we have observed spectral shifts of the emission with propagation distance in both types of fiber, which are greater in the case of the thermosetting fiber. These spectral shifts may be employed to manufacture tunable light sources based on doped POFs. In this sense, we have also shown that wavelength tunability is also possible by increasing the dye concentration in the fiber.

## Figures and Tables

**Table 1:** Manufacturing fiber characteristics.

POF	Type of fiber	Refractive index	Core diameter	Cladding thickness	Dopant concentration	Overlapping factor
Thermoplastic	GI	1.501 *	0.6 mm	0.4 mm	16 ppm **	1.4
Thermosetting	SI	1.54	0.7 mm	0.05 mm	300 ppm	1

* maximum value. ** average value.

**Table 2:** Fluorescence and ASE characteristics of the thermoplastic POF and of the thermosetting one.

POF	Fluorescence		ASE		Threshold energy
	λ_peak_	FWHM	λ_peak_	FWHM	
Thermoplastic	586 nm	40 nm	579 nm	18 nm	0.14 ± 0.02 mJ
Thermosetting	616 nm	47 nm	612 nm	18 nm	0.63 ± 0.02 mJ

**Table 3:** Values of the fittings to the experimental curves of Figure 11a with Equation (3).

POF	*a*	*τ*_1_ (s)	*b*	*τ*_2_ (s)	*R*^2^
Thermoplastic	37.45	113	65.81	47710	0.993
Thermosetting	61.51	1567	43.67	163400	0.999
